# 

**Published:** 1905-08-15

**Authors:** 


### CONTENTS.

Event and Comment - - - - - - - - - - 379

Some Changes in Dental Practice made Necessary by Modern Science, By C. M. Wright, D.D.S 383

How Much Orthodontia Should the General Practitioner Do? By Martin Dewey, D.D.S., M.D. 394

Benefits of Post-Graduate Courses, - - - By A. C. Runyan 399

Practical Treatment of Pyorrhea Alveolaris, By Austin F. James, D.D.S. 404

Desensitizing the Tooth Pulp by Way of the Dentinal Tubules, By J. Edw. Line, D.D.S. 407

Obituary - - - - - - - - - - - - - 415

Indents - - - - - - - - - - - - - 416

Announcements - - - - - - - - - - - 430

